# Successful Treatment of Human Herpesvirus 6 Encephalomyelitis in Immunocompetent Patient

**DOI:** 10.3201/eid1004.030587

**Published:** 2004-04

**Authors:** Eric Denes, Laurent Magy, Karine Pradeau, Sophie Alain, Pierre Weinbreck, Sylvie Ranger-Rogez

**Affiliations:** * Teaching Hospital Dupuytren, Limoges, France

**Keywords:** HHV-6, encephalomyelitis, cidofovir, ganciclovir, adult, immunocompetent, neurologic disease, encephalitis

## Abstract

We report the case of human herpesvirus 6 (HHV-6) encephalomyelitis in an immunocompetent patient, which was confirmed by viral amplification from cerebrospinal fluid. Cidofovir was used followed by ganciclovir because of an adverse effect to probenecid. The patient recovered. HHV-6 should be recognized as one of the causes of encephalomyelitis.

Human herpesvirus 6 (HHV-6) is a member of the *Herpesviridae* family. Like other members of this family, the virus remains in a latent state after primary infection has resolved and can reactivate. HHV-6 encephalomyelitis is an uncommon clinical manifestation in immuncompetent adults. We report the case of a 20-year-old immunocompetent woman who was hospitalized with HHV-6 encephalomyelitis and recovered.

## Case Report

A 20-year-old woman, with no history of medical problems, was admitted to the hospital on February 4, 2002, with a 3-week history of asthenia, myalgia, low-grade fever, urinary retention, and blurred vision. Physical examination showed weakness of all extremities, paresis of her lower limbs, and generalized hyperreflexia. Ocular examination showed a bilateral papillitis and an optic neuritis. The patient was given acyclovir (10 mg/kg, 3x/day) for clinical encephalitis. Despite this treatment, her paresis increased while in the hospital; she was bedridden and unable to sit unsupported. Findings on cranial computed tomographic scan were reported to be normal. Magnetic resonance imaging (MRI) showed a focal lesion in the left thalamus, a medullar cord enlargement, and multiple lesions in the spinal cord white matter. These findings were consistent with inflammatory myelitis but not with multiple sclerosis. The patient did not exhibit any immune abnormalities.

After admission, her first cerebrospinal fluid (CSF) sample was clear with an elevated opening pressure. Its routine analysis indicated 178 leukocytes/mm^3^, with 90% lymphocytes. Total protein and glucose levels were 0.77 g/L and 1.8 mmol/L, respectively. All CSF cultures were negative for bacterial and fungal organisms. The sample was positive for HHV-6 viral DNA by polymerase chain reaction (PCR) (*1*) by using the primers H6.6 (5′-AAGCTTGCACAATGCCAAAAAACAG-3′) and H6.7 (5′-CTCGAGTATGCCGAGACCCCTAATC-3′) amplifying a 223-bp target sequence localized on the open reading frame 13 of HHV-6 and followed by hybridization with the 5R probe (5′-CCGTCTTACTGTATCCGAAACAACTGTCTG-3′), whereas searching for other herpesviruses (i.e, herpes simplex virus type 1 and 2, cytomegalovirus, Epstein-Barr virus, and varicella zoster virus) and enteroviruses by PCR remained negative. HHV-6 was shown to be A type by a previously described typing method (*2*).

Because the patient was deteriorating rapidly, she was given a high dose of intravenous methylprednisolone for 5 days. This treatment was not potent. When the diagnosis of HHV-6 encephalomyelitis was established, methylprednisolone was stopped and cidofovir (5 mg/kg for 1 day) therapy was administered. The patient began to recover, and 6 days after this therapy, results of CSF analysis showed 115 leukocytes/mm^3^, with 95% lymphocytes, a protein level of 0.6 g/L, and negative results of HHV-6 amplification. The patient experienced an adverse skin reaction to probenecid given with cidofovir, and the treatment was stopped. On February 27, the patient was still exhibiting neurologic abnormalities, and her CSF was once again positive for HHV-6 by PCR. Intravenous ganciclovir (5 mg/kg twice daily) was then prescribed for 15 days. Within 1 month, the patient had recovered completely, with no sequelae or abnormalities on MRI. One year after the episode of encephalomyelitis, the patient remained free of neurologic defects (Figure).

**Figure:**
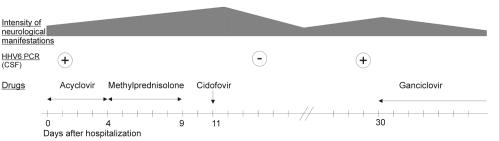
Clinical and therapeutic course and cerebrospinal fluid analysis. Dosage of acyclovir was 10 mg/kg, 3x/day, methylprednisolone was 1 g/day, cidofovir was 5 mg/kg 1 day, ganciclovir was 5 mg/kg, 2x/day.

Several serum samples were taken from the patient on days 2, 12, 22, and 66 after her admission. Serologic tests showed for each serum the same result: anti-HHV-6 immunoglobulin (Ig) G titer of 160 by immunofluorescence assay, accompanied by anti-HHV-6 IgM, except on day 66, showing that the virus had returned to its latent state. The avidity index, measured according to the procedure described by Ward et al. (*3*), was near 100% in the first three serum samples, suggesting that this episode was a reactivation of an existing viral infection. Serologic tests for HIV were repeatedly negative, as were tests for herpes simplex virus, Epstein-Barr virus (EBV), and cytomegalovirus. HHV-6 PCR performed on peripheral blood mononuclear cells was positive, although it was negative in the serum samples.

The same woman was admitted to the emergency room 1 month after discharge because of dysethesia of the lower limbs, tonsillitis, asthenia, and low-grade fever. We assumed it could be a novel reactivation of the HHV-6 infection, but this was not confirmed. It was, in fact, an EBV primary infection. One month later, neurologic manifestations had totally disappeared.

## Conclusions

Humans are widely exposed to HHV-6 during childhood, and the seroprevalence is up to 100% in adults. Two types of HHV-6 (A and B) can be identified; no diseases have clearly been linked to HHV-6A infection, whereas HHV-6B is responsible for the childhood disease exanthem subitum. Exanthem subitum complications, including seizures, hemiplegia, meningoencephalitis, or residual encephalopathy, illustrate HHV-6 neurotropism; HHV-6 commonly invades the brain during ES, even in cases of clinically asymptomatic infections. The virus then persists in brain tissues in a latent form (*4*).

This case is, to our knowledge, the second of encephalomyelitis caused by HHV-6 in an immunocompetent patient. HHV-6 is frequently reported to be implicated in encephalitis or meningoencephalitis in immunocompromised persons, such as HIV-positive patients or transplant recipients, but few reports have implicated HHV-6 in encephalitis in immunocompetent adults (*5*–*7*). Our patient was not immunocompromised by either drug therapy or disease. Serologic tests suggested HHV-6 viral reactivation: IgG were present even in the first serum samples, and the avidity index was high. Tests for IgM were positive as well, but anti-HHV-6 IgM can be found during a viral reactivation (*8*). Symptoms observed were likely to result from a reactivated latent infection of virus in the brain. HHV-6 is known to reactivate frequently during acute infections with other viruses especially with other herpesviruses (*9*). Although our patient had neither obvious immunosuppression nor any confirmed infection, she may have had a selective defect in her responses to HHV-6. The virus can invade the central nervous system and, in some cases, cause acute or subacute encephalitis sometimes associated with diffuse or multifocal demyelinization (*9*).

In other cases of neurologic disease induced by HHV-6, such as encephalomyelitis (*10*), meningoencephalitis (*5*,*6*), or encephalitis (*7*) in immunocompetent adults, patients were treated with acyclovir. Three patients died (*6*,*7*,*9*), and one recovered within 2 days, with small doses of acyclovir (*5*). Data obtained in vitro indicate a greater susceptibility of HHV-6 to cidofovir than ganciclovir or acyclovir (*11*,*12*); acyclovir inhibited viral replication only at high concentrations, so our patient was given cidofovir. This regimen had clinical and virologic efficacy, as the patient started to recover and her CSF improved. Viral DNA was not detectable by PCR 6 days after the first injection of cidofovir. Nevertheless, the patient needed other injections of cidofovir to definitively cure the infection, as shown by HHV-6 DNA in her CSF 16 days after the first injection. The second injection was not possible because of a skin reaction to probenecid. The second treatment given to the patient was ganciclovir, which is known to be effective against HHV-6. On this regimen, the patient completely recovered from HHV-6 encephalomyelitis. Because this is a case report and not a controlled clinical trial, we cannot be certain that the antiviral drugs led to her recovery. We note, however, that after cidofovir therapy was stopped, HHV-6 DNA was again detected in the CSF, concurrent with an increase in neurologic symptoms. She began to recover after starting ganciclovir therapy. As a result, we think that the antiherpesvirus drugs led to her recovery.

Of interest is the EBV primary infection that occurred in this patient 1 month after discharge. The immunosuppression induced by HHV-6 probably favored the EBV infection. The paresis observed during this episode was considered a reactivation of the episode during her HHV-6 infection, since paresis is not a classical manifestation accompanying EBV infection,

In conclusion, the case reported here underlines the fact that HHV-6 may cause rapidly multifocal, demyelinating lesions in an immunocompetent adult, even in the case of viral reactivation. Therefore, we think that HHV-6 should be considered in the differential diagnosis of acute demyelinating encephalomyelitis in immunocompetent adults.
